# High expression circRALGPS2 in atretic follicle induces chicken granulosa cell apoptosis and autophagy via encoding a new protein

**DOI:** 10.1186/s40104-024-01003-w

**Published:** 2024-03-11

**Authors:** Haorong He, Yuanhang Wei, Yuqi Chen, Xiyu Zhao, Xiaoxu Shen, Qing Zhu, Huadong Yin

**Affiliations:** 1 Key Laboratory of Livestock and Poultry Multi-Omics, Ministry of Agriculture and Rural Affairs, College of Animal Science and Technology, Sichuan Agricultural University, Chengdu, Sichuan 611130 China; 2 Farm Animal Genetic Resources Exploration and Innovation Key Laboratory of Sichuan Province, Sichuan Agricultural University, Chengdu, Sichuan 611130 China

**Keywords:** Apoptosis, Autophagy, Chicken, CircRALGPS2, Follicle atresia, PARP1, RNC-seq

## Abstract

**Background:**

The reproductive performance of chickens mainly depends on the development of follicles. Abnormal follicle development can lead to decreased reproductive performance and even ovarian disease among chickens. Chicken is the only non-human animal with a high incidence of spontaneous ovarian cancer. In recent years, the involvement of circRNAs in follicle development and atresia regulation has been confirmed.

**Results:**

In the present study, we used healthy and atretic chicken follicles for circRNA RNC-seq. The results showed differential expression of circRALGPS2. It was then confirmed that circRALGPS2 can translate into a protein, named circRALGPS2-212aa, which has IRES activity. Next, we found that circRALGPS2-212aa promotes apoptosis and autophagy in chicken granulosa cells by forming a complex with PARP1 and HMGB1.

**Conclusions:**

Our results revealed that circRALGPS2 can regulate chicken granulosa cell apoptosis and autophagy through the circRALGPS2-212aa/PARP1/HMGB1 axis.

**Supplementary Information:**

The online version contains supplementary material available at 10.1186/s40104-024-01003-w.

## Background

There are 4–5 million primordial follicles in the chicken ovary at peak, but 99% of these become atretic after follicular selection. Follicular selection directly affects reproductive capacity. Unselected follicles undergo atresia, a process in the ovary that regulates the number of follicles in the developing pool [1, 2]. Abnormal or excessive atresia of follicles can lead to unbalanced follicle development, which in turn results in ovarian disease and abnormal egg production rates. Studies have shown that apoptosis is the root cause of follicular atresia, which begins in the granulosa cell layer when the proportion of apoptotic granulosa cells (GC) in developing follicles reaches more than 10% [3]. Apoptosis is not the only factor contributing to follicular atresia. Recent studies have found that autophagy also mediates follicular growth and atresia [4].

CircRNAs are a class of non-coding RNAs that are widely present in eukaryotic cells. Unlike linear RNAs, the 5' end cap and a 3' end poly(A) tail of circRNAs are covalently bonded to form a closed-loop structure; circRNAs are thus insensitive to RNase and can more stably participate in transcription and post-transcriptional regulation [5]. A large number of studies have shown that circRNAs are closely related to growth and development, stress responses, and disease occurrence in organisms. The biological functions of circRNAs are mainly believed to include transcriptional regulation, miRNA sponging, protein interaction, translation, immunity regulation, and functional complex formation [6].

Although circRNAs are widely reported to control tumorigenesis and myogenesis in humans and animals [6, 7], there are only a few reports on the regulation of reproductive traits, especially in chickens. In the present study, we performed circRNA RNC-seq using healthy and atretic chicken follicles as models to investigate the effects of circRNAs on follicular development and atresia in chickens. Analysis of sequencing data revealed that circRALGPS2 was formed by the circularization of exons 2 to 8 of RALGPS2 and was differentially expressed between healthy and atretic follicles. Furthermore, we identified circRALGPS2 expression in chicken primary granulosa cells in vitro and verified the function of circRALGPS2 in regulating follicle development and atresia through translating proteins. Since chicken is an important model for studying human ovarian diseases [8, 9], our research also provides new insights into the diagnosis and treatment of ovarian dysfunctions in humans, such as human polycystic ovary syndrome (PCOS).

## Methods

### Ethical standards and sample collection

All animal experiments in the present study were approved by the Institutional Animal Care and Use Committee of Sichuan Agricultural University; the approval number is 2019102013. The breed of chicken used in this study was Tianfu broiler, which was provided by the poultry breeding farm of Sichuan Agricultural University, Ya’an, Sichuan, China.

Follicles used for circRNA translation sequencing were obtained from the 200-day-old Tianfu broilers. The healthy small yellow follicles were obtained from three egg-laying chickens, and atretic small yellow follicles were obtained from three brooding chickens. The yolk was squeezed out from the granular layer of the follicles, and the follicles were then washed gently with phosphate-buffered saline (PBS, Hyclone, Logan, UT, USA). Additionally, heart, liver, spleen, lung, kidney, uterus, ovary, breast muscle, adipose, and cerebrum tissues were collected from the three egg-laying chickens. All collected samples were immediately frozen in liquid nitrogen and stored in a –80 °C freezer.

### CircRNA RNC-seq analysis

A circRNA RNC-seq analysis was performed by Gene Denovo Biotechnology Co., Ltd. (Guangzhou, Guangdong, China). Follicles were immediately frozen in liquid nitrogen and triturated in liquid nitrogen before being dissolved in 400 μL of lysis buffer. This extract was then immediately loaded onto a 1 mol/L sucrose pad prepared in polysome buffer containing 0.1 U/mL SuperaseIn™ RNase (Invitrogen, Carlsbad, CA, USA). The ribosomes were then pelleted in a TLA-110 rotor by centrifugation for 4 h. The liquid supernatant was removed, and the pellet was resuspended in 10 mmol/L Tris and immediately mixed with SDS. The samples obtained were then heated to 65 °C, and RNA was extracted using two rounds of acidic phenol/chloroform treatment. After total RNA extraction, rRNA was removed using previously reported methods [10]. The enriched mRNAs and ncRNAs were fragmented into short fragments using fragmentation buffer and reverse transcribed into cDNA using random primers. Next, the cDNA fragments were purified using the QIAquick PCR extraction kit (Qiagen, Venlo, The Netherlands), end repaired, affixed with a poly(A) tail, and ligated to Illumina sequencing adapters. The second strand cDNA was then digested using UNG (uracil-N-glycosylase). The digested products were size-selected using agarose gel electrophoresis, PCR-amplified, and sequenced using an Illumina HiSeq^TM^ 4000 instrument obtained from Gene Denovo Biotechnology Co., Ltd.

To obtain highly clean reads, the reads were further filtered using FASTP (version 0.18.0). The short read alignment tool Bowtie2 (2.2.8) was used to map reads to a ribosomal RNA (rRNA) database and the rRNA mapped reads were then deleted. The remaining reads were further used for transcriptome assembly and analysis. The reference genome was indexed, and paired-end clean reads were mapped to the reference genome using HISAT2.2.4. Mapped reads for each sample were assembled through a reference-based method using RSEM v1.3.1. For each transcribed region, FPKM (transcript fragments per kilobase per million mapped reads) values were calculated to quantify its expression abundance and variation. The FPKM formula used is as follows:

$$FPKM=\frac{1{0}^{6}c}{NL/1{0}^{3}}$$

Differential translation analysis of RNA between two groups was performed using the DESeq2 software, while differential translation analysis of RNA between two samples was performed using the edgeR software. The genes with the a false discovery rate (FDR) below 0.05 and absolute fold change 2 were considered as differentially translated genes (DTGs). The DTGs were then subjected to GO function and KEGG pathway enrichment analyses.

CircRNA RNA-seq analysis on the same samples was performed by Novogene Technology Co., Ltd. (Beijing, China). Products were purified and library quality was assessed on an Agilent Bioanalyzer 2100 system. Index-encoded samples were clustered on the cBot Cluster Generation System using the TruSeq PE Cluster Kit v3-cBot-HS (Illumina, San Diego, CA, USA) according to the manufacturer’s instructions. After cluster generation, the library was sequenced on the Illumina Hiseq 4000 platform and 150 bp paired-end reads were generated (SRA accession number: PRJNA721929).

### Cell culture and transfection

The granulosa layers of follicles were isolated using the Gilbert method [11]. For the detailed procedure of the collection of chicken primary granulosa cells, please refer to our previous report [12]. The medium used for culturing granulosa cells contained 10% fetal bovine serum (FBS, Gibco, Langley, OK, USA) and 40% medium 199 (m199, Gibco). Cells were incubated at a constant temperature of 37 °C, 5% CO_2_, and saturated humidity. The culture time of primary granulosa cells depended on the different experiments but did not exceed one week. Primary granulosa cells were round or oval in the initial stage, but after 48 h of culture, the cells began to grow and took on a polygonal or spindle shape. FSHR, as a granulosa cell-specific protein, is expressed and localized in the cytoplasm of granulosa cells [13]. We identified the cells we collected as primary granulosa cells by FSHR immunofluorescence [12]. The chicken DF-1 cell line was used for the dual-luciferase reporter assay and circRNA identification.

The RNA oligonucleotides and overexpression plasmids were designed according to the sequences of circRALGPS2, PARP1, and HMGB1 (Table S1). The vector pcD25-ciR was used to construct a circRNA overexpression plasmid, and the vector pcDNA-3.1 was used to construct a gene overexpression plasmid. All RNA oligonucleotides were constructed by GenePharma (Shanghai, China), and all overexpression plasmids were constructed by Beijing Tsingke Biotech Co., Ltd. When cells reached 60%–80% confluency, RNA oligonucleotides and overexpression plasmids were immobilized in cells using Lipofectamine 3000 (Invitrogen) and Opti-MEM (Gibco).

### Total RNA extraction and real-time quantitative PCR

Total RNA was extracted from cells using TRIzol reagent (Invitrogen), according to the manufacturer’s instructions. The circRNAs and mRNAs were then reverse transcribed using Prime Script RT Master Mix Perfect Real Time (Takara, Dalian, Liaoning, China), while miRNAs were reverse transcribed using a one-step miRNA cDNA synthesis kit (HaiGene, Harbin, Heilongjiang, China). The primers for real-time qPCR were designed using the Premier 6 software (Premier Biosoft, San Francisco, CA, USA). The specific information of primers is presented in Table S2. Three replicates of real-time qPCR were performed per sample. Each reaction mixture included 1 μL cDNA, 0.5 μL reverse primer, 0.5 μL forward primer, 3 μL double distilled water, and 5 μL TB Green™ Premix Ex Taq™ II (Takara). The β-actin gene and *U6* were used as internal controls.

### CircRNA identification

Primers for circRALGPS2 were designed, and the PCR amplification products were subsequently subjected to Sanger sequencing (Sangon Biotech, Shanghai, China). Total RNA was extracted and then treated with RNase R (Lucigen, Middleton, WI, USA), according to the manufacturer’s instructions at 37 °C for 10 min; this was followed by RNase R inactivation at 90 °C for 10 min. RT-qPCR was then used to detect circRALGPS2 and β-actin resistance to RNase R. The total RNA was reverse transcribed into cDNA using random primers (N9) and oligo-d(T)_18_ primers (Transgen, Beijing, China). The cDNAs obtained by reverse transcription of total RNA with random primers and oligo-d(T)_18_ primers were used for RT-qPCR to analyze the efficiencies of different reverse transcription primers and further analyze the circular structure characteristics of circRALGPS2.

### Immunohistochemistry (IHC)

For immunohistochemical analysis, the paraffin-embedded tissue sections on slides were first deparaffinized. The slides were then sequentially washed in xylene, absolute ethanol, 85% alcohol, 75% alcohol, and distilled water. Next, the slides were placed in EDTA antigen retrieval buffer (Servicebio, Wuhan, Hubei, China) and incubated in a microwave oven for antigen retrieval. After natural cooling of the slide-containing buffer, the slides were washed with shaking on a destaining shaker; the slides were then placed in 3% hydrogen peroxide solution, incubated at room temperature in the dark, and washed with shaking on a destaining shaker. After blocking with serum (Solarbio, Beijing, China), the sections were incubated with a primary antibody overnight at 4 °C. The next day, the slides were washed with shaking on a destaining shaker, and the sections were then incubated at room temperature for 50 min. After the sections were slightly dried, freshly prepared DAB chromogenic solution (Servicebio) was added to them dropwise. Harris hematoxylin (Servicebio) was added for counterstaining for about 3 min. The slides were then sequentially placed in 75% alcohol, 85% alcohol, absolute ethanol, and xylene to be dehydrated till transparent. Cover slips and neutral gum were then used to seal the sections. Finally, a fluorescence microscope (Olympus, Melville, NY, USA) was used to examine and photograph the sealed sections.

### Western blotting

The total protein of the cells was extracted according to the instructions of the total protein extraction kit (BestBio, Shanghai, China) manufacturer. Protein concentrations were then normalized using the BCA protein assay kit (BestBio), following the manufacturer’s instructions. For the specific steps of the Western blotting procedure, refer to our previous study [12]. Details of the antibodies used in the experiments are provided in Table S3.

### Nuclear-cytoplasmic fractionation and fluorescence in situ hybridization (FISH)

Nuclei and cytoplasm were isolated according to the PARIS™ Kit (Invitrogen) manufacturer’s instructions. FISH probes for circRALGPS2 (cy3) was synthesized by GenePharma Co., Ltd. FISH probes for circRALGPS2 were designed to span the splice sites of circRALGPS2. The FISH probe sequences are GATCAT + TCCTAAATAGGGAA + TACATGG. Chicken GC cells were collected and used to perform FISH using an RNA FISH kit (GenePharma) according to the manufacturer’s instructions. Finally, probed-target localization was observed under a confocal microscope (Olympus).

### IRES activity assay and luciferase reporter assay

According to the RNA secondary structure of circRALGPS2, different plasmids were constructed using the Luc2-IRES-Reporter vector (Geneseed). The Luc2-IRES-Reporter vector plasmid and dual-luciferase reporter gene were constructed and synthesized by Beijing Tsingke Biotech Co., Ltd. Chicken DF1 cells were then collected and transfected with the plasmids. Next, luciferase activity was detected using a dual-luciferase reporter gene detection kit (Beyotime, Shanghai, China). The luminescent activities of firefly luciferase and Renilla luciferase were finally detected using a multi-plate reader (Biotek, Winooski, VT, USA), according to the manufacturer’s instructions.

### Immunoprecipitation (IP) and LC-MS analysis

After transfection of ov-circRALGPS2-FLAG into chicken granulosa cells, IP experiments were performed according to the manufacturer instructions of the Pierce™ Co-Immunoprecipitation Kit (Thermo Scientific, Waltham, MA, USA). For specific steps of the procedure, refer to our previous research [14]. The protein complexes collected after IP were separated using sodium dodecyl sulfate–polyacrylamide gel electrophoresis (SDS-PAGE) and analyzed using Western blotting and LC-MS/MS.

The protein gel after SDS-PAGE was enzymatically hydrolyzed and decolorized using test staining solution/silver staining solution. The obtained extracts were then combined and vacuum-dried. Zip-tip desalination was then performed. Next, peptide samples were diluted to 1 μg/μL on the cabinet. The sampling volume was set to 5 μL and scanning was then performed for 60 min. The samples were scanned for peptides with a mass-to-charge ratio of 350−1,200. Mass spectral data were collected using a Triple TOF 5600+ LC/MS system (AB Sciex, Foster City, CA, USA). Peptide samples were then analyzed using a Triple TOF 5600 plus mass spectrometer coupled to an Eksigent nanoLC system (AB Sciex). The MS spectrum was scanned using IDA (Information Dependent Acquisition) at an ion accumulation time of 250 ms.

The MS/MS raw files from the mass spectrometer were submitted to ProteinPilot (https://sciex.com.cn/products/software/proteinpilot-software, version 4.5; Sciex, Redwood City, CA, USA) for data analysis. Protein identification was performed using the Paragon algorithm in ProteinPilot with certain filtering criteria. Peptides with an unused score > 1.3 (over 95% confidence) were considered credible peptides, and proteins containing at least one unique peptide were retained.

### Confocal microscopy

The mcherry-EGFP-LC3 adenovirus was purchased from Hanbio (Shanghai, China). Following interference or overexpression of non-coding RNAs and mRNAs in granulosa cells, adenoviruses were diluted and transfected into the cells. For detailed steps of the procedure, refer to the previous study [12]. The cells were then observed and photographed under a confocal microscope (Olympus).

### Flow cytometric analysis

The apoptosis of granulosa cells was detected using flow cytometry. Apoptosis detection and data analysis were performed using the CytoFLEX flow cytometer (Beckman Coulter) and Kaluza 2.1 software (Beckman Coulter), respectively. For specific steps, refer to the previous study [12].

### Statistical analysis

Statistical analysis of data was performed using the SPSS 19.0 statistical software (SPSS, Inc., Chicago, IL, USA). Comparative analysis between the two groups was performed using unpaired Student's *t-*test. Multiple groups were compared using one-way ANOVA and are marked with the letters a, b, c, d, e, and f. Differences in all statistical tests were considered significant when *P* < 0.05 and extremely significant when *P* < 0.01. All data are presented as the least squares standard error of the mean (SEM). Each experiment was performed in at least three biological replicates.

## Results

### Apoptosis and autophagy are enhanced during follicular atresia

**Fig. 1:**
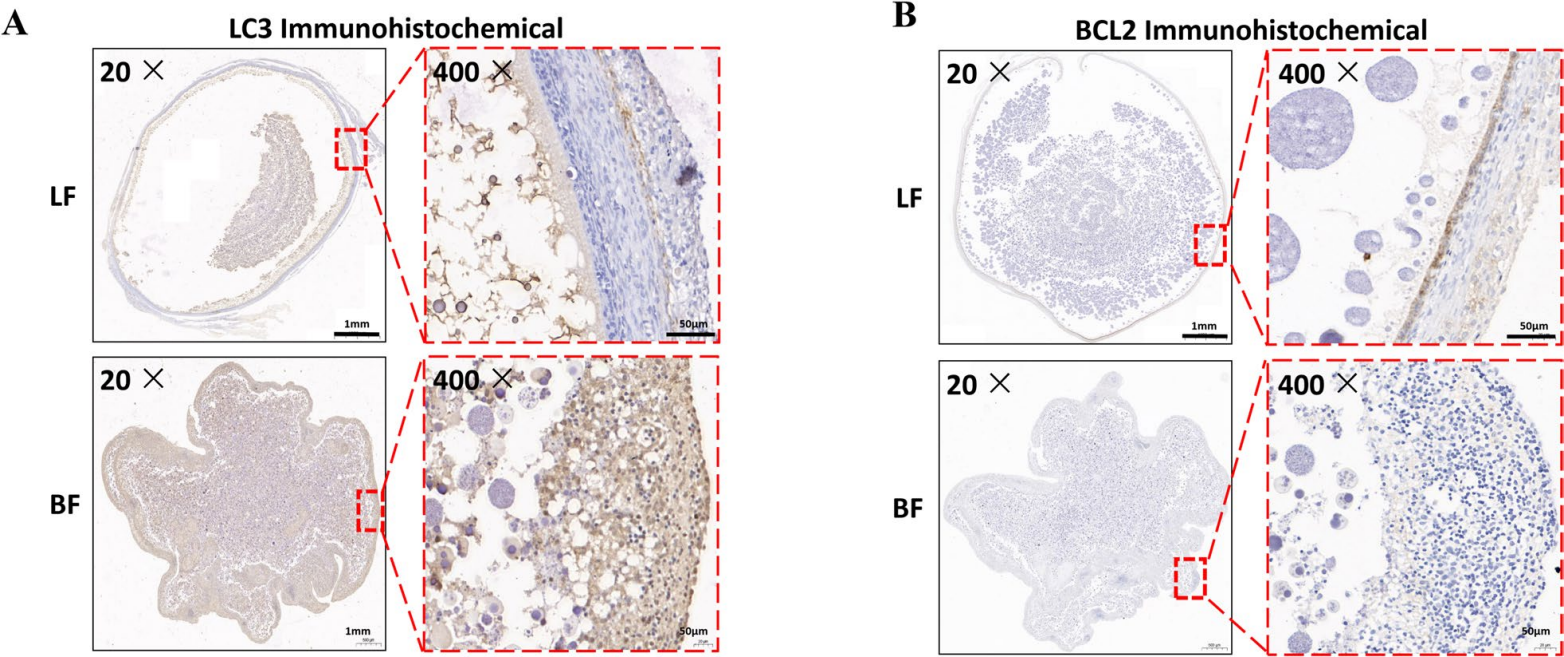
Apoptosis and autophagy are enhanced during follicular atresia. **A** Immunohistochemistry of healthy and atretic follicles with BCL2 antibodies. **B** Immunofluorescence of healthy and atretic follicles with BCL2 antibodies

Our previous study found that the ovaries were atrophied and the follicles were shrunken during follicular atresia [12, 15]. Moreover, there are follicles of different development stages in normal ovaries, including grade follicles and pre-grade follicles. The brooding chicken has a large number of white follicles and a small number of small yellow follicles, and has bleeding symptoms. In the present study, immunohistochemistry results demonstrated that LC3 (Cell Signaling Technology, #12741) levels were significantly up-regulated and BCL2 (Biorbyt, #orb100697) levels were significantly down-regulated (Fig. 1A and B) compared with basal levels in follicular atresia.

### CircRNA RNC-seq analysis results of healthy and atretic follicles

**Fig. 2:**
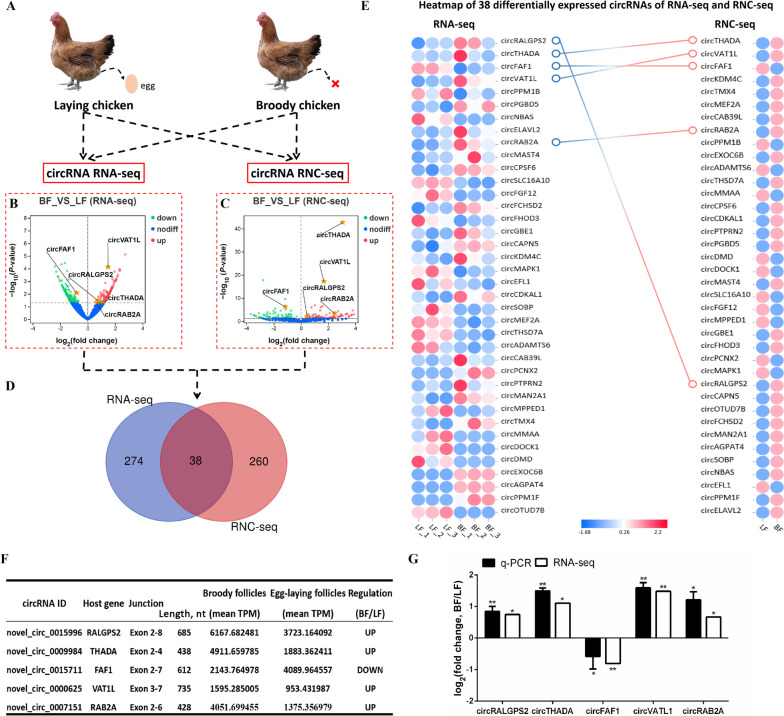
CircRNA RNC-seq analysis of healthy and atretic follicles. **A** Schematic sequencing of healthy and atretic follicles. **B** Volcano plot of differentially expressed circRNAs in circRNA RNC-seq. **C** Volcano plot of differentially expressed circRNAs in circRNA RNA-seq. **D** Combined analysis of circRNA RNA-seq and RNC-seq. **E** Heatmap of 38 differentially expressed circRNAs in circRNA RNA-seq and RNC-seq. **F** Details of the top 5 circRNAs with the highest expression derived from exon splicing. **G** qRT-PCR validation of five dierentially expressed circRNA in healthy and atretic follicles. ^*^P < 0.05, ^**^P < 0.01. Data are presented as mean ± SEM. *n* = 9 for qRT-PCR

Following the circRNA RNC-seq analysis of healthy and atretic follicles (Fig. 2A), all raw sequencing data was stored in the SRA database (Accession number: PRJNA858499). The Q20 and Q30 sequencing quality values of RNC-seq were greater than 97.87% and 93.82% (Table S4). After enriching polyA tail-containing mRNAs using Oligo(dT) magnetic beads, the retained unmapped reads were used for translational analysis (Table S5). Most circRNAs were derived from the splicing of exons and a few were derived from the splicing of introns and intergenic regions (Fig. S1A). Differential analysis revealed 297 differentially expressed circRNAs, of which 157 were highly expressed in atretic follicles (Fig. 2B). The GO enrichment analysis results of differentially expressed circRNAs showed that the biological processes involving proteins expressed by them included cell proliferation, cell killing, and cellular process (Fig. S1B). The KEGG enrichment analysis showed that the main processes involving proteins encoded by differentially expressed circRNAs included transcription, translation, cell growth and death, autophagy-animal, and apoptosis. In addition, the major signal transduction pathways included the TGF-β signaling pathway, mTOR signaling pathway, Wnt signaling pathway, and insulin signaling pathway (Fig. S1C).

RNA-seq (SRA accession number: PRJNA721929) provided 51.85 Gb of clean bases. Moreover, 311 differentially expressed circRNAs were revealed between healthy and atretic follicles, 164 circRNAs had higher expression in atretic follicles and 147 circRNAs had lower expression in atretic follicles (Fig. 2C). The length distribution of circRNAs showed that the full circRNA length was negatively correlated with the number of circRNAs of that length, and most circRNAs had a full length below 10,000 nt (Fig. S2A). Most identified circRNAs were derived from the splicing of exons and a few were derived from the splicing of introns and intergenic regions (Fig. S2B). The GO enrichment results showed that biological processes involving identified circRNAs included the execution phase of apoptosis, cellular component disassembly, autophagy, and negative regulation of autophagy (Fig. S2C). The KEGG enrichment analysis showed that the main signal transduction pathways involving the source genes included the TGF-beta signaling pathway, mTOR signaling pathway, MAPK signaling pathway, and insulin signaling pathway (Fig. S2D).

Combined with data from circRNA RNA-seq and RNC-seq. We found that thirty-eight differentially expressed circRNAs were both identified (Fig. 2D). Cluster analysis showed that the expression patterns of circRNAs sequenced using RNA-seq and RNC-seq were similar (Fig. 2E). We selected the five most differentially expressed circRNAs derived from exon splicing for subsequent validation (Fig. 2F) and found that their expression patterns were the same as those revealed by RNA-seq (Fig. 2G).

### Identification of circRALGPS2

**Fig. 3:**
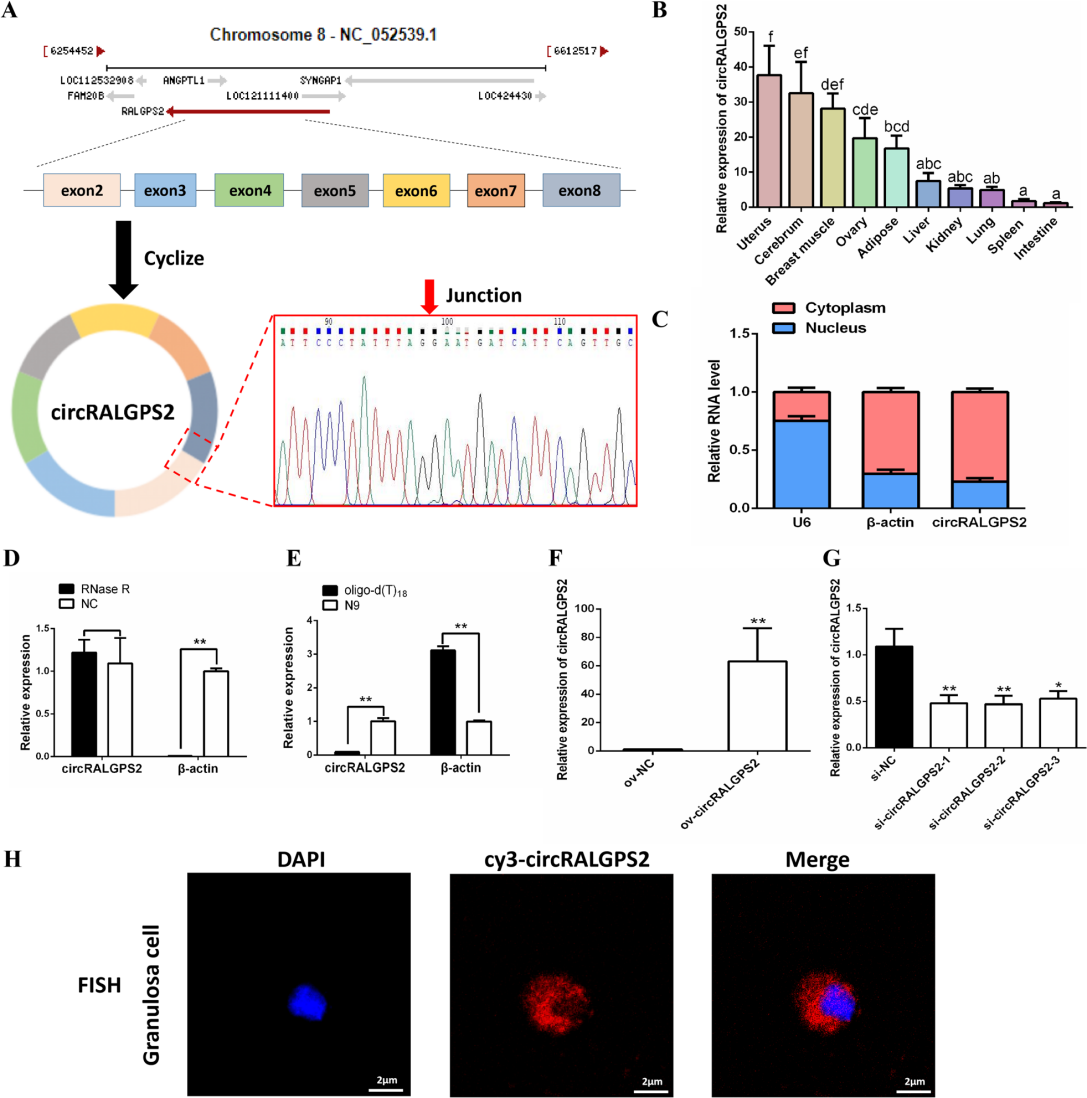
Identification of circRALGPS2. **A** Schematic diagram of the source of circRALGPS2 and Sanger sequencing results. **B** Expression of circRALGPS2 in 10 different chicken tissues. **C** Expression levels of circRALGPS2 in the cytoplasm (red) and nucleus (blue) of granulosa cells. **D** Expression of linear RNA and circRALGPS2 after RNase R treatment. **E** qRT-PCR of linear RNA and circRALGPS2 from cDNA reverse transcribed from oligo-d(T)_18_ and random primers. **F** Expression of circRALGPS2 after transfection with ov-circRALGPS2 plasmid. **G** Screening of different si-circRALGPS2 fragments. **H** FISH assay of circRALGPS2 in chicken granulosa cells. ^a–f^Bars with different letters indicate significant differences (*P* < 0.05), ^*^P < 0.05, ^**^P < 0.01. Data are presented as mean ± SEM. *n* = 9 for qRT-PCR

Sequence analysis revealed that circRALGPS2 was formed by the splicing of exons 2 to 8 of the RALGPS2 gene, which has back-site junctions (Fig. 3A). Moreover, circRALGPS2 was highly expressed in reproductive organs, such as the uterus and ovary (Fig. 3B). The subcellular localization of circRALGPS2 in chicken granulosa cells was found to mainly be the cytoplasm (Fig. 3C). Next, the RNase R resistance test showed that circRALGPS2 was more resistant to RNase R digestion than linear RNA (β-actin) (Fig. 3D). Besides, we were able to amplify circRALGPS2 only through cDNA reverse transcribed using random primers (N9), but not cDNA reverse transcribed using oligo-d(T)_18_ (Fig. 3E). These results indicated that circRALGPS2 is a stable circular RNA. The cytoplasmic localization of circRALGPS2 was also confirmed by constructing a FISH probe of circRALGPS2 and obtaining immunofluorescence images after overexpressing ov-circRALGPS2 in granulosa cells (Fig. 3C and H, and Fig. S3). Finally, we successfully constructed si-circRALGPS2-2 and ov-circRALGPS2 vectors for subsequent functional verification experiments (Fig. 3F and G).

### Determination of the protein-coding ability of circRALGPS2

**Fig. 4:**
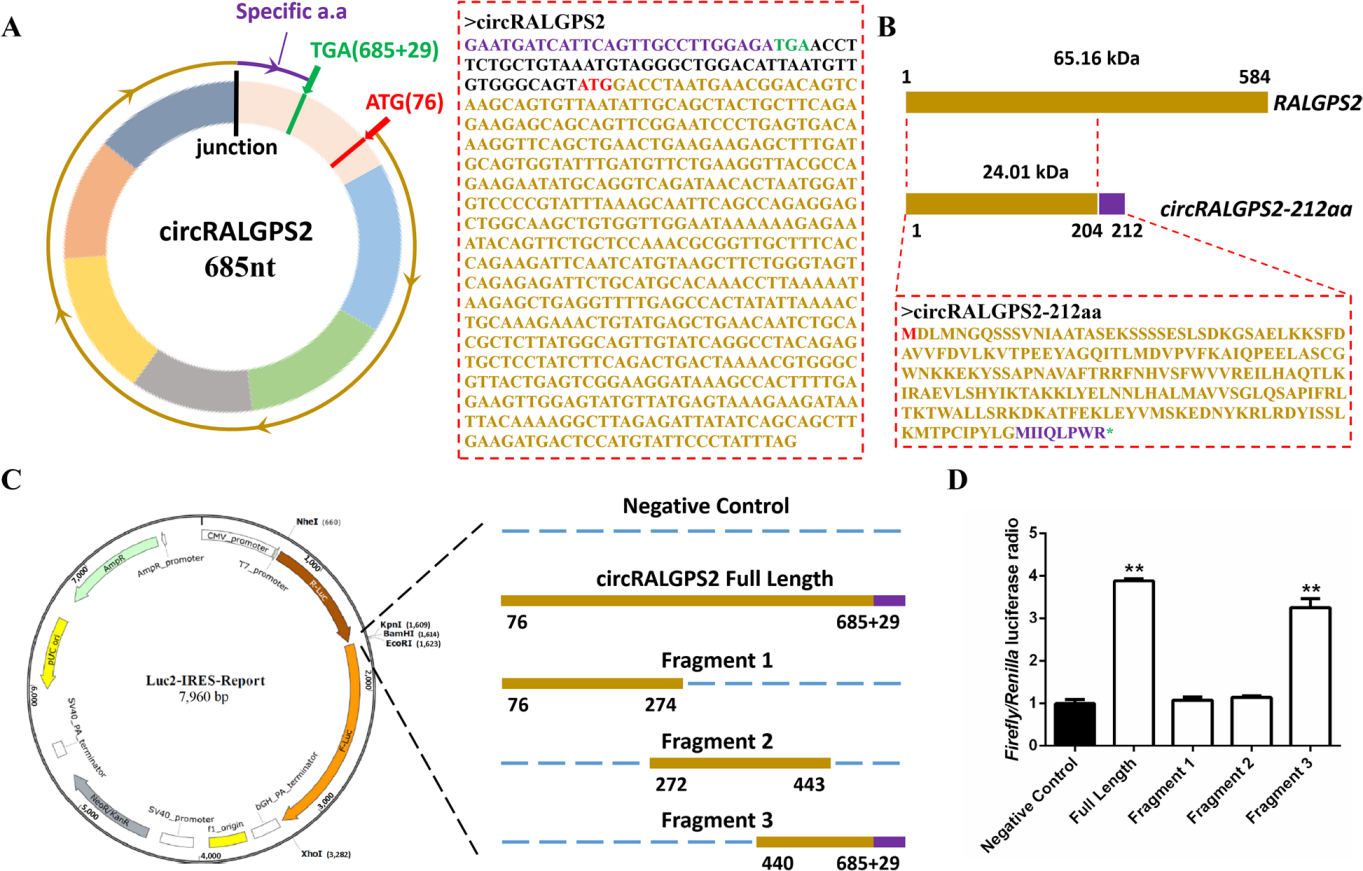
Protein translation prediction of circRALGPS2. **A** Schematic representation of the open reading frame of circRALGPS2. **B** Schematic diagram of circRALGPS2 translation protein circRALGPS2-212aa, the purple fragment is the specific peptide of circRALGPS2-212aa. **C** Schematic diagram of a dual-luciferase reporter gene (Luc2-IRES-Reports vector) containing different fragments of circRALGPS2 for IRES validation. **D** The ratio of firefly luciferase to Renilla luciferase activities after transfection of the dual-luciferase reporter vector into DF-1 cells. ^**^P < 0.01. Data are presented as mean ± SEM. *n* = 3 for Double luciferase reporting test

CircRALGPS2 contains a non-conserved ORF that could encode a 212 amino acid protein (circRALGPS2-212aa) with a predicted molecular weight of 24.01 kDa; the translation of this proteins would start from exon 2 of RALGPS2 and end at exon 2 after crossing the circRALGPS2 splice site (Fig. 4A). Unlike the amino acid sequence of RALGPS2, circRALGPS2-212aa contains a deliberate amino acid sequence (MIIQLPWR*; Fig. 4B).

To validate the presence of an IRES in circRALGPS2, its secondary structure was first analyzed; three fragments that could possibly contain IRES were obtained (Fig. S4). Full-length circRALGPS2 and these three fragments were then inserted into the Luc2-IRES-Reporter vector to construct plasmids (Fig. 4C). The luciferase activity revealed that full-length circRALGPS2 significantly enhanced the F-Luc luciferase activity, indicating that circRALGPS2 contains an IRES-like sequence. Moreover, circRALGPS2 fragment 3 significantly enhanced the F-Luc luciferase activity, suggesting that this fragment contains an IRES-like sequence (Fig. 4D).

### Identification of a 212-amino acid (aa) novel protein encoded by circRALGPS2

**Fig. 5:**
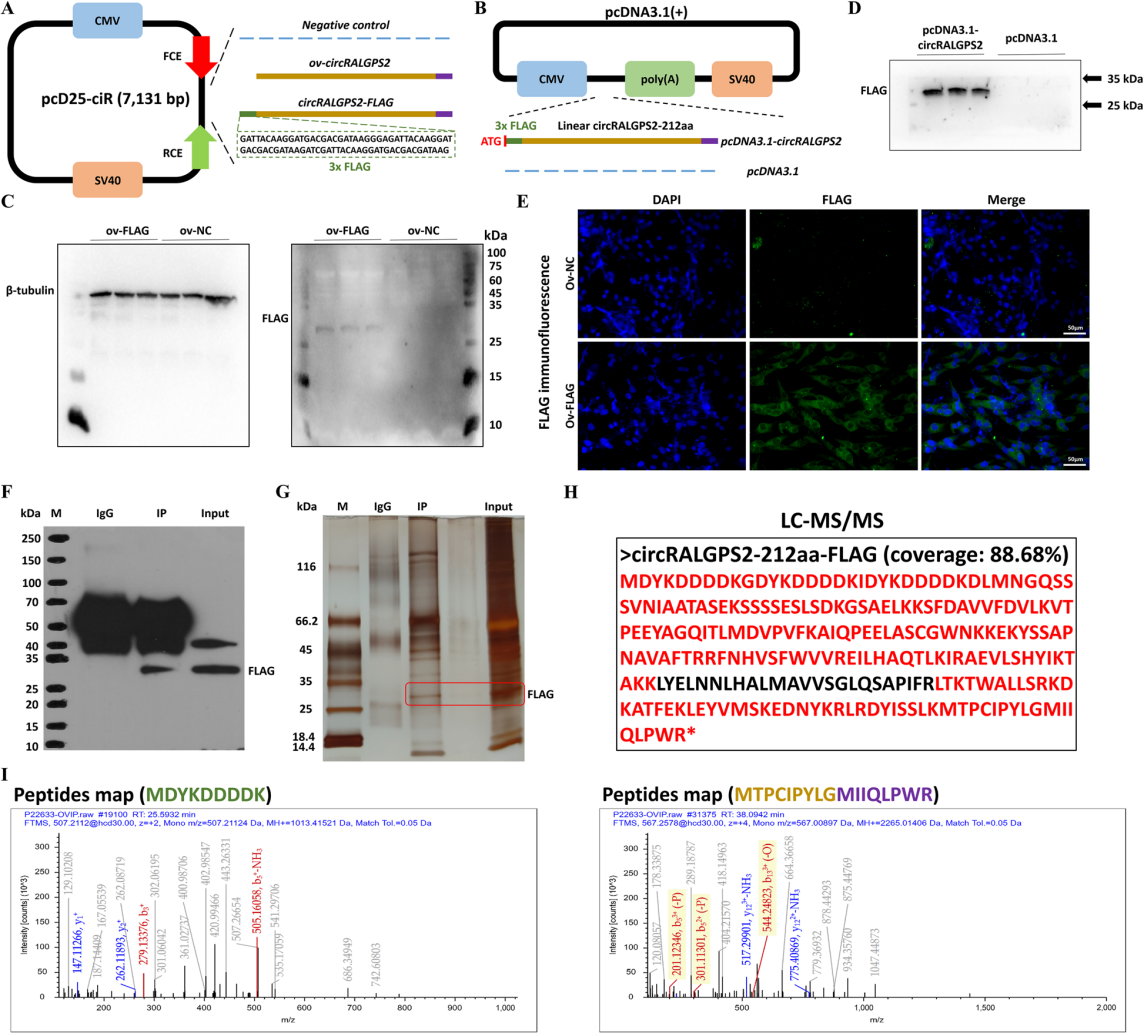
Identification of circRALGPS2-212aa. **A** Schematic diagram of the construction of ov-circRALGPS2 and ov-circRALGPS2-FLAG. **B** Schematic diagram of the construction of pcDNA3.1-circRALGPS2-FLAG. **C** Levels of Flag-tagged protein and β-tubulin protein after transfection of ov-circRALGPS2-FLAG in granulosa cells. **D** Flag-tagged protein levels after transfection of pcDNA3.1-circRALGPS2-FLAG in granulosa cells. **E** Immunofluorescence of Flag-tagged after transfection of ov-circRALGPS2-FLAG in granulosa cells. **F** Western blot results of co-immunoprecipitation with FLAG antibody in granulosa cells. **G** Silver staining assay results after co-immunoprecipitation with FLAG antibody in granulosa cells. **H** LC-MS/MS analysis of circRALGPS2 peptides after co-immunoprecipitation. **I** LC-MS/MS analysis of inserted FLAG peptides and specific peptides of circRALGPS2

Since the predicted circRALGPS2-212aa was a novel protein, there was no specific antibody against it that could be used for its analysis. Therefore, we inserted a 3× FLAG sequence into circRALGPS2 to obtain ov-circRALGPS2-FLAG (Fig. 5A), which encoded a 27.16 kDa protein. The linear sequence of circRALGPS2-212aa FLAG-tagged at the start codon was inserted into the pCDNA3.1 plasmid (Fig. 5B). Western blotting using an anti-FLAG antibody detected a 25–35 kDa protein in the ov-circRALGPS2-FLAG transfection group but not in the ov-NC transfection group (Fig. 5C). Similarly, a 25–35 kDa protein was detected using a FLAG antibody after pcDNA3.1-circRALGPS2 transfection (Fig. 5D). Immunofluorescence results confirmed that circRALGPS2-212aa expression was increased after tagging it with the 3× FLAG sequence (Fig. 5E). Co-immunoprecipitation in DF-1 cells transfected with ov-circRALGPS2-FLAG using FLAG and IgG antibodies showed an obvious protein band at the 25–35 kDa size for the input group and the IP-FLAG group. This result was completely consistent with the predicted size of the protein encoded by circRALGPS2-212aa-FLAG; however, there was no corresponding protein band in the IP-IgG group (Fig. 5F and G). The IP-FLAG eluate was further analyzed by LC-MS/MS, and 94 amino acids of the circRALGPS2-212aa-FLAG protein sequence were identified, accounting for 88.68% of the total sequence (Fig. 5H and Table S6), including the 3× FLAG sequence and the specific amino acid sequence (MIIQLPWR) of circRALGPS2-212aa (Fig. 5I). Taken together, these results confirmed that circRALGPS2 can translate into circRALGPS2-212aa.

### CircRALGPS2-212aa promoted chicken granulosa cell apoptosis and autophagy

**Fig. 6:**
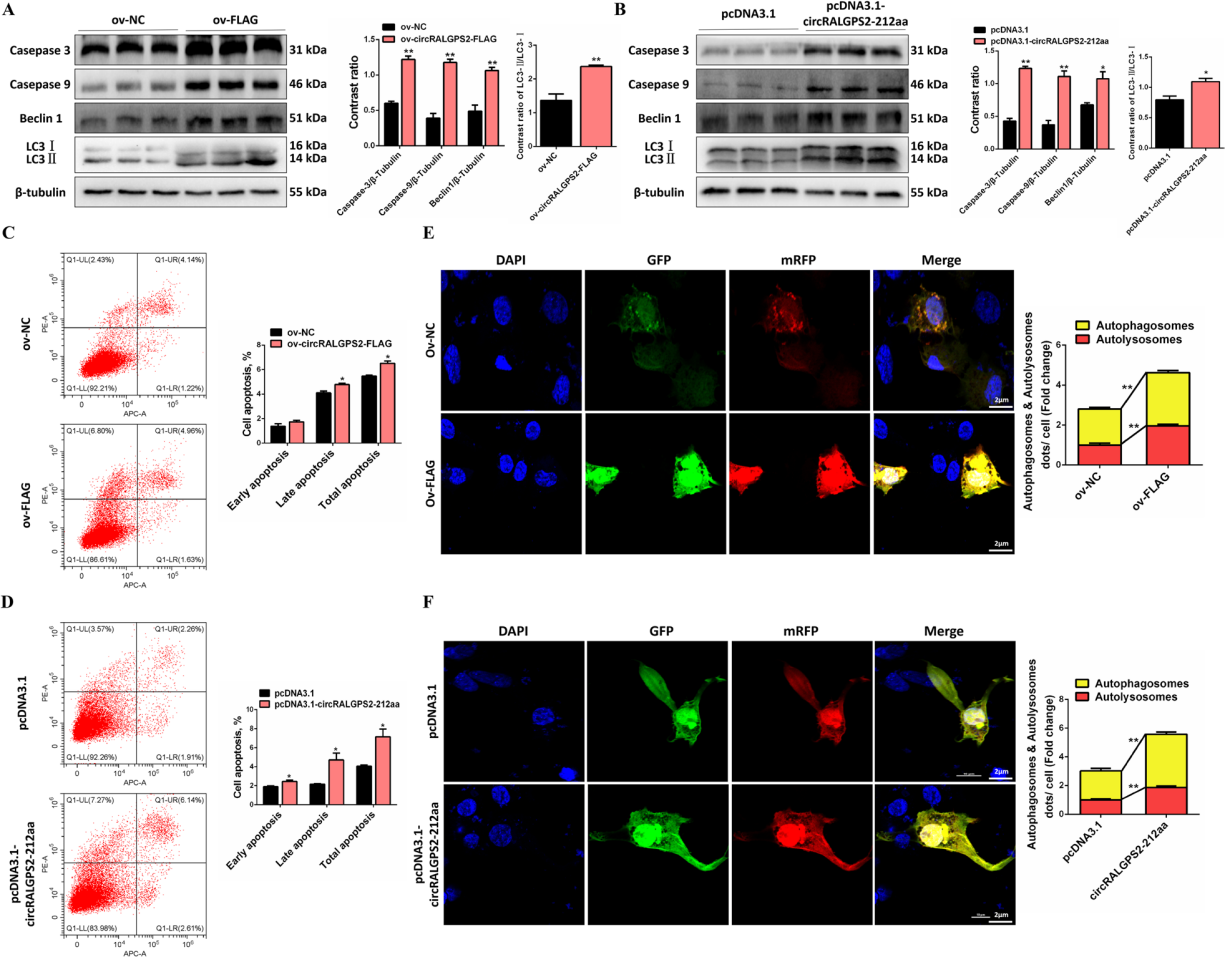
CircRALGPS2-212aa promoted chicken granulosa cell apoptosis and autophagy. **A** The protein abundance and gray value statistics of caspase 3, caspase 9, Beclin 1 and LC3 in granulosa cells after transfection with ov-circRALGPS2-FLAG plasmid. **B** The protein abundance and gray value statistics of caspase 3, caspase 9, Beclin 1 and LC3 in granulosa cells after transfection with pcDNA3.1-circRALGPS2-FLAG plasmid. **C** Flow cytometry results of granulosa cells after transfection with ov-circRALGPS2-FLAG plasmid. **D** Flow cytometry results of granulosa cells after transfection with pcDNA3.1- FLAG plasmid. **E** Confocal microscopy observation of autophagosomes in granulosa cells after transfection with ov-circRALGPS2-FLAG plasmid. Green fluorescence, lysosome; red fluorescence, autophagosome; yellow fluorescence, autophagosome. **F** Confocal microscopy observation of autophagosomes in granulosa cells after transfection with pcDNA3.1-circRALGPS2-FLAG plasmid. ^*^P < 0.05, ^**^P < 0.01. Data are presented as mean ± SEM. *n* = 9 for qRT-PCR, and *n* = 3 for Western blotting

To investigate the effect of circRALGPS2-212aa on the apoptosis and autophagy of granulosa cells in chickens, the GCs were transfected with the ov-circRALGPS2-FLAG and pcDNA3.1-circRALGPS2 plasmids in vitro. Western blotting results confirmed that the expression levels of caspase 3, caspase 9, and Beclin 1 proteins increased and the conversion of LC3-I to LC3-II accelerated after GC transfection with ov-circRALGPS2-FLAG (*P* < 0.05, Fig. 6A). After transfection of GCs with the pcDNA3.1-circRALGPS2 plasmid, the abundance of caspase 3, caspase 9, and Beclin 1 proteins also increased and the conversion of LC3-I to LC3-II also accelerated (*P* < 0.05, Fig. 6B). The flow cytometry results confirmed that circRALGPS2-212aa could promote granulosa cell apoptosis (*P* < 0.05, Fig. 6C and D). Contrarily, results obtained after mcherry-EGFP-LC3 adenovirus infection showed that transfection with the ov-circRALGPS2-FLAG plasmid or pcDNA3.1-circRALGPS2 plasmid increased the number of autophagosomes (Fig. 6E and F). Taken together, these findings prove that the circRALGPS2-212aa protein has a positive effect on granulosa cell apoptosis and autophagy.

### CircRALGPS2-212aa protein interacts with PARP1 protein

**Fig. 7:**
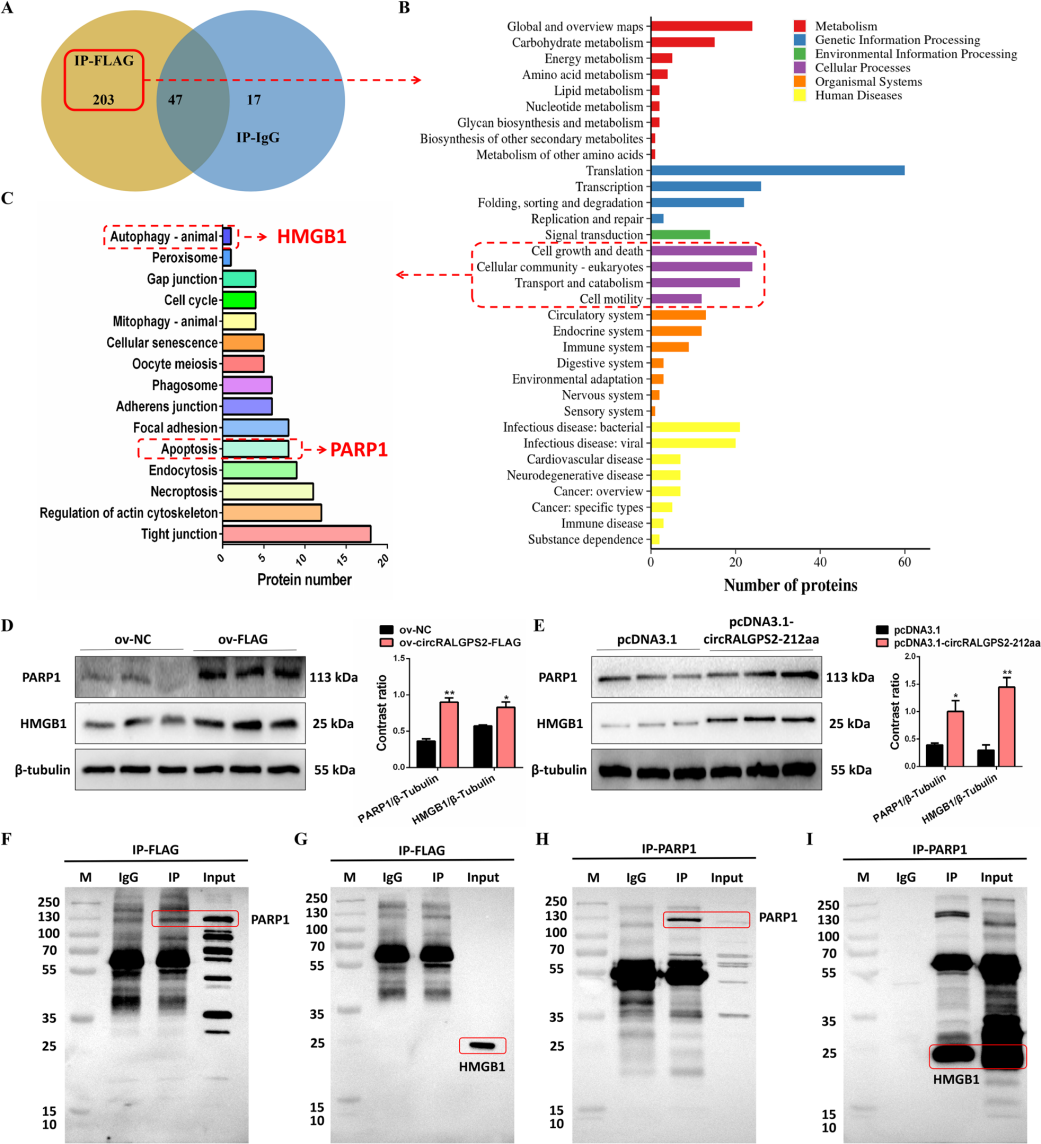
CircRALGPS2-212aa protein interacts with PARP1 protein. **A** Proteins identified by co-immunoprecipitation with FLAG antibody in granulosa cells. **B** and **C** KEGG enrichment analysis of identified proteins. **D** The protein abundance and gray value statistics of PARP1 and HMGB1 after transfection with ov-circRALGPS2-FLAG plasmid. **E** The protein abundance and gray value statistics of PARP1 and HMGB1 after transfection with pcDNA3.1-circRALGPS2-FLAG plasmid. **F** Western blot results of PARP1 antibody after granulosa cells were co-immunoprecipitated with FLAG antibody. **G** Western blot results of HMGB1 antibody after granulosa cells were co-immunoprecipitated with FLAG antibody. **H** Western blot results of PARP1 antibody after granulosa cells were co-immunoprecipitated with PARP1 antibody. **I** Western blot results of HMGB1 antibody after granulosa cells were co-immunoprecipitated with PARP1 antibody. ^*^P < 0.05, ^**^P < 0.01. Data are presented as mean ± SEM. *n* = 3 for Western blotting

The LC-MS/MS analysis identified 250 proteins in the IP-FLAG eluate and 64 proteins in the IP-IgG eluate; 47 proteins were commonly identified in the two eluates (Fig. 7A and Table S7). After removing the 47 commonly identified proteins, KEGG enrichment analysis of the functions of the remaining 203 proteins identified using IP-FLAG was performed; the results showed that one protein (HMGB1) was associated with autophagy-animals and eight proteins (including PARP1) were associated with apoptosis (Fig. 7B and C, and Table S8). After transfection with the ov-circRALGPS2-FLAG plasmid or pcDNA3.1-circRALGPS2 plasmid, the protein abundances of PARP1 and HMGB1 in circRALGPS2-212aa-overexpressing GC cells were increased (Fig. 7D and E). After co-immunoprecipitation of circRALGPS2-212aa, PARP1 was detected in both the eluate and the lysate (Fig. 7F), but HMGB1 was only detected in the lysate (Fig. 7G). Additionally, we found that PARP1 could form a complex with HMGB1 through co-immunoprecipitation and Western blotting (Fig. 7H and I).

### PARP1 promotes apoptosis and HMGB1 promotes autophagy in chicken granulosa cells

**Fig. 8:**
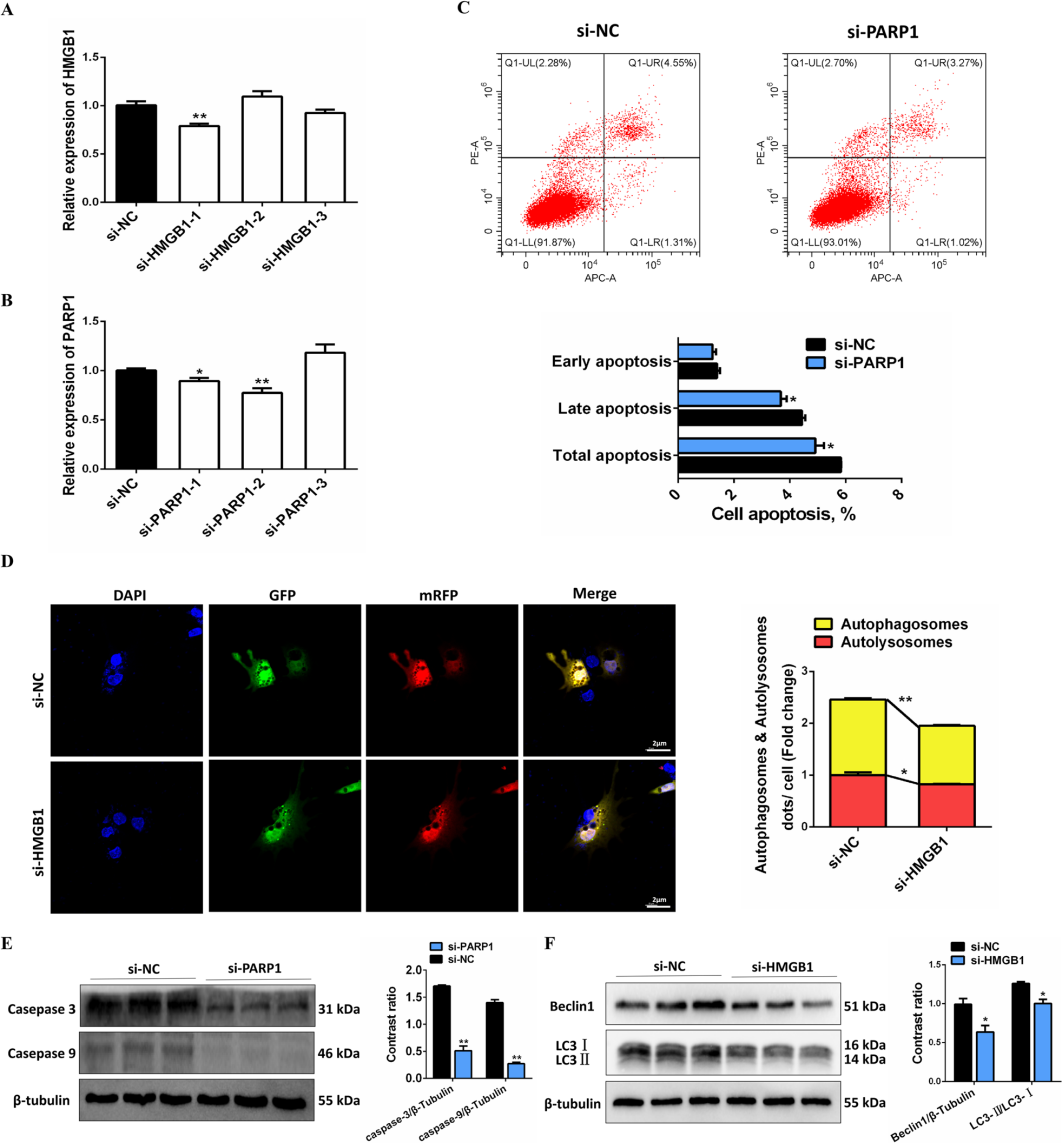
PARP1 promotes apoptosis and HMGB1 promotes autophagy in chicken granulosa cells. **A** Screening of different si-PARP1 fragments. **B** Screening of different si-HMGB1 fragments. **C** Flow cytometry results in granulosa cells after interference with PARP1. **D** Confocal microscopy observation of autophagosomes in granulosa cells after transfection with si-HMGB1. **E** The protein abundance and gray value statistics of caspase 3 and caspase 9 after transfection with si-PARP1. **F** The protein abundance and gray value statistics of Beclin 1 and LC3 after transfection with si-HMGB1. ^*^P < 0.05, ^**^P < 0.01. Data are presented as mean ± SEM. *n* = 9 for qRT-PCR, and *n* = 3 for Western blotting

We next investigated whether circRALGPS2-212aa interacts with PARP1 and HMGB1 to regulate granulosa cell apoptosis and autophagy. For this, we first synthesized siRNAs that could inhibit PARP1 and HMGB1 (Fig. 8A and B). Then, flow cytometry and Western blotting found that the apoptosis rate and protein abundance of caspase 3 and caspase 9 in GCs decreased after PARP1 knockdown (Fig. 8C and D). Additionally, the mcherry-EGFP-LC3 adenovirus infection and Western blotting results showed that the number of autophagosomes, Beclin 1 protein abundance, and LC3-II/LC3-I ratio decreased after HMGB1 knockdown (Fig. 8E and F). Taken together, these results indicated that circRALGPS2-212aa promotes apoptosis and autophagy by directly forming a complex with PARP1 in chicken GCs.

## Discussion

In the recent years, research on the circRNA-mediated regulation of important economic traits of domestic animals has gradually emerged. For example, there has been progress in research regarding circRNA involvement in the regulation of muscle growth and development, providing a reference for the comprehensive analysis of the regulatory mechanism of muscle development [16, 17]. The regulation of ovarian development by circRNAs has also been reported in a variety of animals, such as mouse [18], pig [19], cattle [20], sheep [21], and bee [22]. In particular, a study on the characterization of granulosa cell circRNA expression profiles in healthy and atretic antral follicles of pigs found that abnormally expressed circRNAs may play a role in follicular atresia [23]. In the present study, we selected healthy follicles and atretic follicles as sequencing models for circRNA RNA-seq and RNC-seq. Regarding the fact that only 10% of circRNA DEGs are identical between the two methods, it may be due to differences in sequencing methods. RNC-seq extracts ribosomal RNA by centrifugation and detects circRNA combined with ribosomal RNA. RNA-seq is different, ribosomal RNA is first removed, and rRNA-free residues are removed through ethanol precipitation. Subsequently, linear RNA was digested with RNase R, and then circRNA was measured. Because only a small part of circRNA translates into proteins that can bind to ribosomal RNA. Of course, although the samples are the same, the two sequencing were completed by two companies, and the different instruments used may be the reason for the large difference. Through combined analysis of sequencing data, 38 differentially expressed circRNAs were found between healthy follicles and atretic follicles, among which circRALGPS2, formed by RALGPS2 exons 2–8, had the highest expression. According to a previously conducted circRNA transcriptome sequencing of chicken yellow small follicles (SYF, 5–8 mm), smallest-grade follicles (F6, 9–12 mm), and largest-grade follicles (F1, ~ 40 mm), circRALGPS2 was differentially expressed among different developmental stages of chicken follicles [24]. According to the research of Shen et al. [24], the expression of circRALGPS2 in SYF, F6 and F1 follicles showed a downward trend. Due to the strong early follicular selection, it is suggested that RALGPS2 circular RNA may be a factor promoting atresia. This is consistent with our conclusion. At the same time, Shen verified 3 circRALGPS2 isoforms, and the translatable circRALGPS2 we found is one of the 3 isoforms they verified (circRNA_8:6,369,673|6,402,248). These findings suggest that circRALGPS2 may play a regulatory role in chicken follicular development.

It is generally recognized that non-coding RNAs (ncRNAs) exist widely and mostly cannot be translated into proteins. But emerging evidence has confirmed that some so-called ncRNAs, including long noncoding RNAs (lncRNAs) and primary miRNAs (pri-miRNAs), can produce functional peptides in vivo [25, 26]. In the recent years, similar reports have been found for circRNAs. In 2017, ribosome imprinting in *Drosophila* heads demonstrated that a set of circRNAs is associated with ribosomes involved in protein translation and proved that UTRs of ribo-circRNAs (cUTRs) allow cap-independent translation [27]. In the same year, Legnini et al. [28] found that circ-ZNF609 contains an open reading frame and can be translated into protein in a splicing-dependent and capsid-independent manner, providing proof that circRNAs can encode proteins in eukaryotes. Subsequently, more and more studies have been conducted on circRNAs capable of being translated into proteins. To date, several circRNA-encoded proteins have been found to play key roles in cancers, including colon cancer, breast cancer, and liver cancer [29–31]. In agricultural animals, Wang et al. found that circEgg encodes a protein circEgg-p122 that could inhibit histone H3 lysine 9 trimethylation (H3K9me3) while promoting H3 lysine 9 acetylation through miR-3391-5p sponging in silkworms in 2020 [32]. Next, a previous study conducted by us in poultry found that the novel protein circFAM188B-103aa, encoded by circFAM188B, promoted the proliferation of chicken skeletal muscle satellite cells but inhibited their differentiation [14]. Current research on circRNA has found that the mechanism by which circRNA participates in protein coding mainly occurs in the cytoplasm, and circRNA formed by gene exon splicing is mainly expressed in the cytoplasm [6]. To prove that circRALGPS2 is a circRNA formed by exon splicing, subcellular localization of circRALGPS2 was performed, including nucleocytoplasmic isolation of granulosa cells and FISH. The results showed that circRALGPS2 was mainly located in the cytoplasm of granulosa cells. Next, the results of the present study robustly demonstrate that circRALGPS2 expressed in chicken follicles can encode a novel protein named circRALGPS2-212aa. We then confirmed that this protein can promote granulosa cell apoptosis and autophagy. It is worth noting that the role of RALGPS2 on granulosa cells is unclear. According to our results, after transfection of ov-circRALGPS2-FLAG, WB only detected proteins of 25–35 kDa size, and no expression of the parent gene RALGPS2 (65.16 kDa) was detected. We can preliminarily rule out the influence of RALGPS2 protein. But it still needs to be verified through more precise experiments in the future.

We found through further functional studies of circRALGPS2-212aa that it can interact with PARP1 and HMGB1. Based on our KEGG enrichment analysis, PARP1 was found to be involved in the apoptosis pathway and HMGB1 was found to be involved in the autophagy pathway. Next, we confirmed that PARP1 promoted cell apoptosis and HMGB1 stimulated cell autophagy in vitro. In the results, the silencing efficiency of PARP1 and HMGB1 was not very high, which may be related to the transfection dose and time. Of course, it may also be related to the primary cells or siRNA design. The silencing efficiency of the genes we previously verified is not always satisfactory [33]. But overall, statistically the impact of interference does exist. PARP1 is a ribozyme that is activated upon DNA damage and balances apoptosis and necrosis by regulating cellular ATP levels [34]. In addition, the activation of PARP1 can promote autophagy by inhibiting AMPK and mTOR signaling [35]. HMGB1 has been widely reported to play a central role in the induction of autophagy by upregulating the expression of HSP27 in the nucleus, activating the Beclin-1/PI3K-III complex in the cytoplasm, meanwhile, and extracellularly binding with receptors to form advanced glycation end products [36]. As for co-IP result, IgG has bands, but they are obviously much weaker than those of the IP group. We suspect that it may be due to reasons such as the detergent concentration in the lysis buffer being too high or the formula being too vigorous, or the interaction between protein and protein being too weak or unstable. Therefore, subsequent experiments were conducted to verify whether circRALGPS2 has an effect on PARP1. The results of the present study indicated that circRALGPS2-212aa can form a complex with PARP1 but cannot directly interact with HMGB1. However, we also found that PARP1 can directly interact with HMGB1, consistently with findings of previous reports indicating that PARP1 can bind to HMGB family proteins to play a regulatory role [37, 38]. The abovementioned results suggest that the mechanism through which circRALGPS2-212aa regulates granulosa cell apoptosis and autophagy involves its binding to PARP1 and the activation of HMGB1, filling the information gap regarding the involvement of circRNA-encoded proteins in domestic animal reproduction.

## Conclusions

**Fig. 9:**
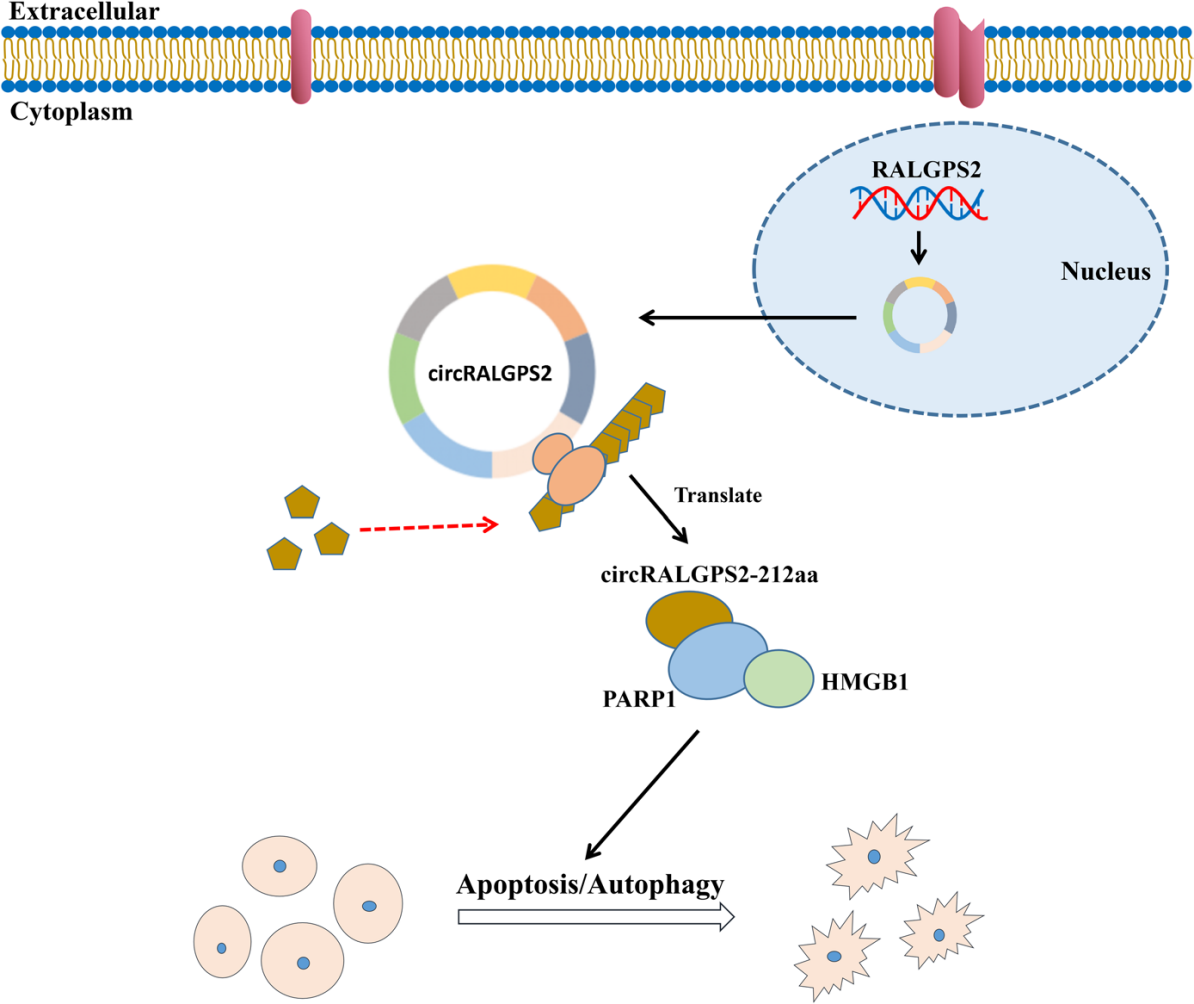
Mechanisms of circRALGPS2 regulating follicle development and atresia in chickens

The results of the present study indicated that circRALGPS2 regulates follicle development and atresia in chickens by promoting the apoptosis and autophagy of granulosa cells via the circRALGPS2-212aa/PARP1/HMGB1 axis (Fig. 9). These findings clarify the molecular mechanisms underlying follicular development and atresia in chicken. Chicken is an important model for the study of human ovarian diseases; therefore, the results of the present study will also enable the development of strategies for the diagnosis and treatment of ovarian dysfunctions in humans.

### Supplementary Information

**Additional file 1:** **Fig. S1.** CircRNA RNC-seq analysis of healthy and atretic follicles. **Fig. S2.** CircRNA RNA-seq analysis of healthy and atretic follicles. **Fig. S3.** FISH assay of circRALGPS2 in chicken granulosa cells (200x). **Fig. S4**. The RNA secondary structure analysis of circRALGPS2.

**Additional file 2:** **Table S1.** RNA oligonucleotides in article. **Table S2.** Primers used for quantitative real-time PCR. **Table S3.** Antibodies used in article. **Table S4.** Summary of the quality of RNC-seq data output. **Table S5.** Alignment removes ribosomes in RNC-seq. **Table S6.** Amino acid sequence of circRALGPS2-212aa-FLAG protein identified by LC-MS/MS analysis. **Table S7.** Proteins identified after LC-MS/MS analysis. **Table S8.** KEGG enrichment analysis results of 203 proteins identified by IP-FLAG.

## Data Availability

The circRNA RNA-seq and RNC-seq raw data were stored in the SRA database, and the accession number were PRJNA721929 and PRJNA858499 (https://www.ncbi.nlm.nih.gov/sra). The mass spectra data were provided in Table S6–8.

## Abbreviations

ATG5: Autophagy related 5
ATG7: Autophagy related 7
CircRNAs: Circular RNAs
DF-1: Chicken embryo fibroblasts cell line
DMEM: Dulbecco’s Modified Eagle Medium
FBS: Fetal bovine serum
FLAG: Flagellar protein
FSH: Follicle-stimulating hormone
GCs: Granulosa cells
GO: Gene Ontology
HMGB1: High mobility group box 1
HPG axis: Hypothalamus-pituitary-gonad axis
HRP: Horseradish peroxidase
IRES: Internal ribosome entry site
KEGG: Kyoto Encyclopedia of Genes and Genomes
LH: Luteinizing hormone
PARP1: Poly(ADP-ribose) polymerase 1
PBS: Phosphate buffered saline
PI: Propidium iodide
RALGPS2: Ral GEF with PH domain and SH3 binding motif 2
RNase R: Ribonuclease R
RNC-seq: Ribosome Nascent-chain Complex sequencing
SEM: Standard error of mean
SiRNA: Small interfering RNA
TPM: Transcripts per million
NC: Negative control
