# Supplementary material for: High expression circRALGPS2 in atretic follicle induces chicken granulosa cell apoptosis and autophagy via encoding a new protein
Source: J Anim Sci Biotechnol. 2024 Mar 11;15:42. doi: 10.1186/s40104-024-01003-w (PMC10926623; doi:10.1186/s40104-024-01003-w)
Supplement: Supplementary file 1 — Additional file 1: Fig. S1. CircRNA RNC-seq analysis of healthy and atretic follicles. Fig. S2. CircRNA RNA-seq analysis of healthy and atretic follicles. Fig. S3. FISH assay of circRALGPS2 in chicken granulosa cells (200x). Fig. S4. The RNA secondary structure analysis of circRALGPS2. [file 40104_2024_1003_MOESM1_ESM.pdf]

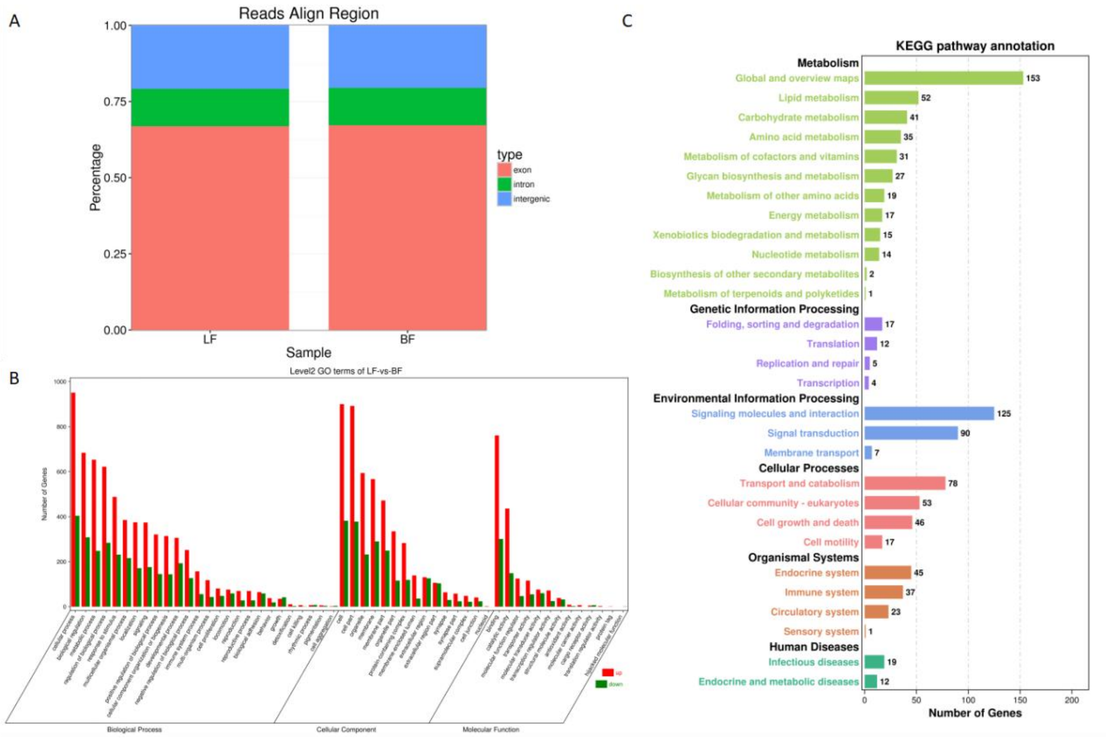

Figure S1. circRNA RNC-seq analysis of healthy and atretic follicles. (A) Source of circRNAs for all samples. (B) Gene Ontology enrichment of source genes of differentially expressed circRNAs. (C) KEGG enrichment of source genes of differentially expressed circRNAs.

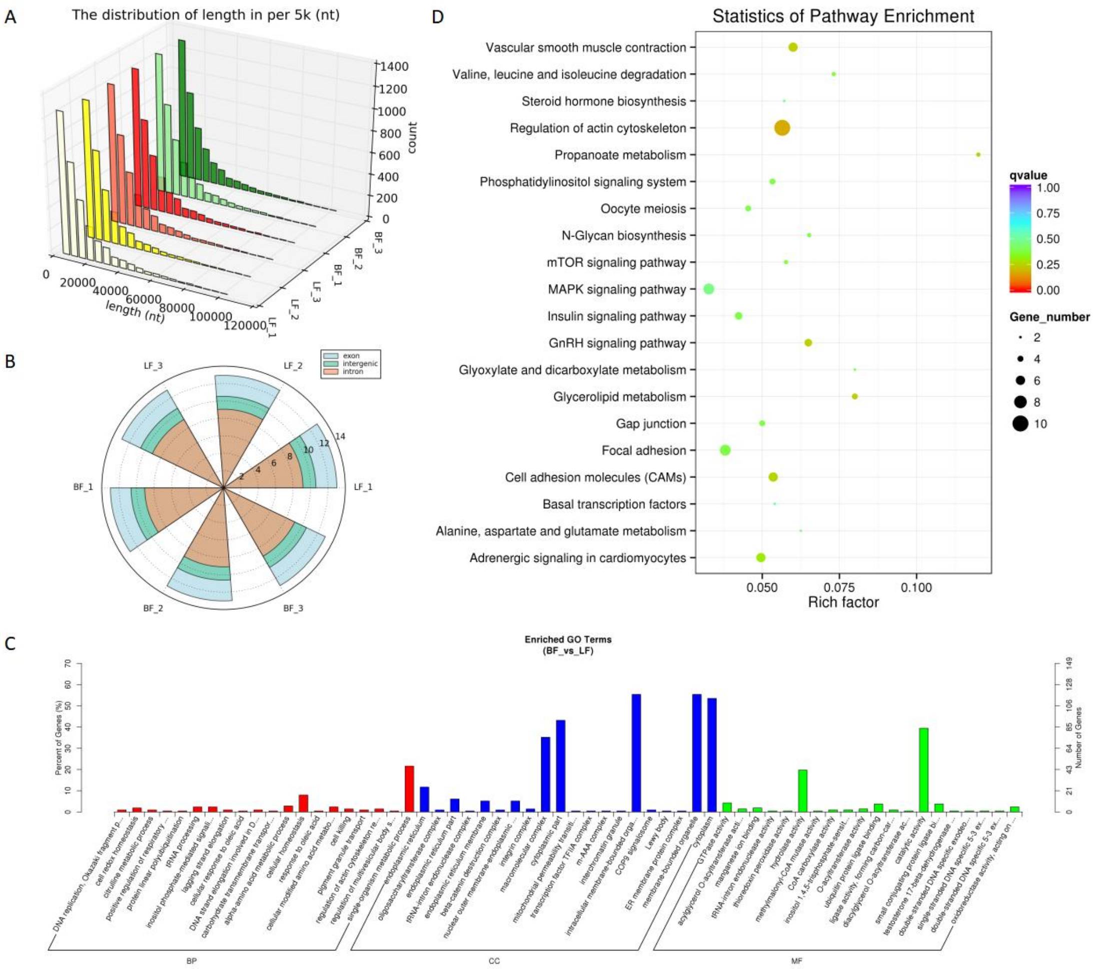

Figure S2. circRNA RNA-seq analysis of healthy and atretic follicles. (A) Length distribution of circRNAs across all samples. (B) Source of circRNAs for all samples. (C) Gene Ontology enrichment of source genes of differentially expressed circRNAs. (D) KEGG enrichment of source genes of differentially expressed circRNAs.

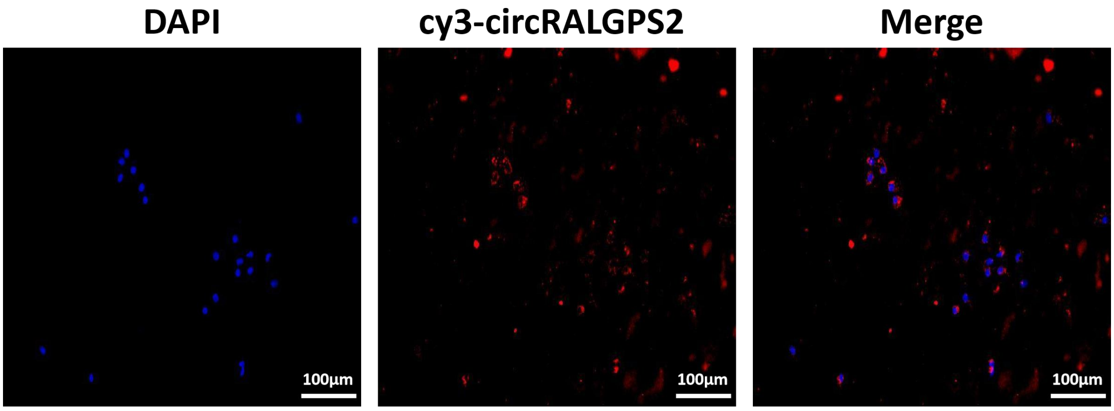

Figure S3. FISH assay of circRALGPS2 in chicken granulosa cells (200 ×).

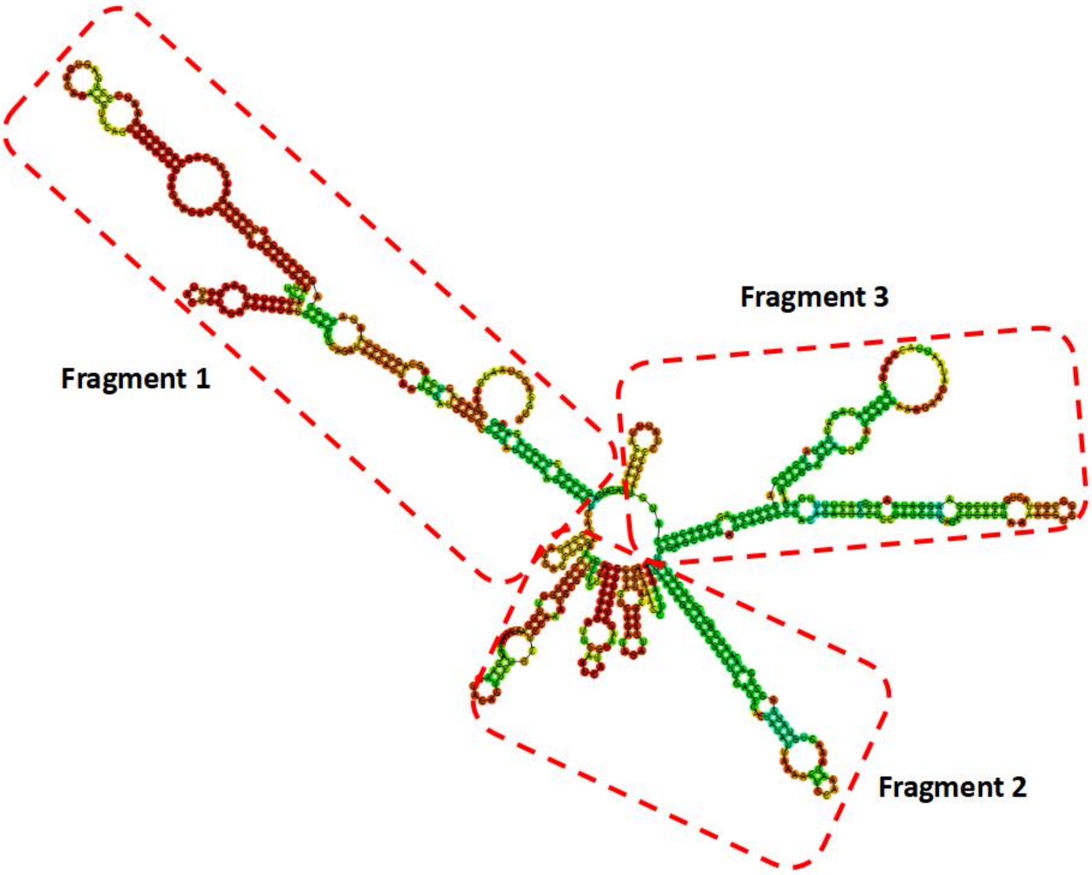

Figure S4. The RNA secondary structure analysis of circRALGPS2. The linear sequence of circRALGPS2 were divide into 3 fragments according to the secondary structure of RNA.
